# The Barancik award lecture: Multi-disciplinary research will be the key to stop, restore, and end MS

**DOI:** 10.1177/13524585251314756

**Published:** 2025-01-28

**Authors:** Sergio E Baranzini

**Affiliations:** Department of Neurology, Weill Institute for Neurosciences, University of California San Francisco, San Francisco, CA, USA

**Keywords:** Genetics, microbiome, disease prediction, machine learning

## Abstract

The past 25 years have brought extraordinary advances in our understanding of MS pathogenesis and the subsequent development of effective therapies. Collaborative genetics efforts have uncovered the association of 236 common DNA variants with disease susceptibility and the first association with disease severity, paving the way to more effective therapies, particularly for progressive forms of the disease. In parallel, and in addition to established environmental disease triggers or modifiers, new collaborative work has revealed new associations with components of the gut microbiome. This research opened a new and exciting prospect for exploring the gut–brain axis, with the potential to also provide new pharmacologic targets and diet-based therapies. Finally, with the availability of massive amounts of information and unprecedented computer power, a new wave of artificial intelligence (AI)-based research is sprawling. These investigations will result in statistically powerful predictive models to identify individuals at risk even years before the disease is clinically apparent. Furthermore, using approaches like semantic representation and causal inference, some of these approaches will be explainable in biomedical terms, thus making them trusted and facilitating their implementation in the clinical setting. The common thread that characterizes all of these advances is multi-disciplinary collaboration among scientists in the form of formal consortia, working groups, or ad hoc partnerships. This may be the “secret sauce” of modern science and the best strategy to stop, restore, and end MS.

## Multiple sclerosis is complex

Multiple sclerosis (MS) is an autoimmune disease of the central nervous system (CNS). This means it affects arguably the two most complex systems in the human body. An emerging consensus among scientists is that the CNS (comprised of the brain and the spinal cord) represents the most complex structure in the known universe.^1^ With approximately 100 billion neurons, each potentially connected to 10,000 other neurons in an intricate architecture, the paths of electrical signals traveling through the CNS appear indescribable. Yet, it reproducibly responds every time we want to move a limb, open our eyes, or learn a new task.

The immune system displays a different kind of complexity. In terms of numbers, it contains an even larger number of cells.^2^ While the brain is “wired” in incredibly elaborated patterns, the 1.8 trillion cells of the immune system move frantically up and down our bloodstream, patrolling every organ and keeping us safe from potential pathogens.

Thus, when we try to understand a disease that affects the brain and the immune system, it is not an understatement to say that it may be the most complex disease ever described. To address this challenge, the global MS research community has coalesced around three main objectives: to stop, restore, and end MS.^3^ The general consensus is that collaborative, multi-disciplinary work will most effectively accelerate progress toward increased knowledge, effective treatments, improved health outcomes and, ultimately, cures for MS. In this article, I describe the efforts my lab has led or contributed to advance research that brings these goals one step closer.

## Genetics

The pattern of familial aggregation of MS cases, together with the higher concordance between identical (i.e. monozygotic) twins, led to the identification of the human histocompatibility antigen (HLA) system as the first genetic association with MS in the early 1970s.^4^ However, despite intense efforts, no additional genetic associations were identified in the three decades that followed.^5–8^ These unsuccessful efforts could have been interpreted as there being no additional genetic factors responsible for MS susceptibility. Alternatively (and most likely), much larger sample sizes would be required for these studies to reach the statistical power needed to identify the expected smaller effects of additional, non-HLA associations. Realizing that individual efforts were unlikely to gather the number of research subjects necessary to unequivocally address this question, leading scientists from those previous efforts agreed to work together, and the International MS Genetics Consortium (IMSGC) was constituted in the early 2000s. Shortly after, the first genome-wide association study (GWAS) was conducted on 1000 trios (affected, mother, and father), using the relatively novel method of transmission disequilibrium test (TDT), which identified common alleles that were most frequently transmitted from parents to patients.^9^ This study uncovered the genomic regions near IL2Ra and IL7Ra genes, the first two MS-associated loci outside the previously known HLA region.^10^ In the following years, smaller studies were also conducted with insufficient power to uncover additional associations convincingly.^11–14^ However, a large meta-analysis confirmed the previously identified loci and added common DNA variants near TNFRSF1A, IRF8, and CD6 to the roster of MS-susceptibility factors.^15^

While identical twins (developing from the same split embryo) share their germline genomes, they still display minor differences in their DNA, mostly as a consequence of accumulated somatic mutations, recombination in immune-related genes (antibodies and T-cell receptors (TCRs)), and epigenetic events.^16^ Determining whether these differences could account, at least in part, for the significant discordance in disease prevalence among monozygotic twins was an unattainable goal until the early 2000s. The Human Genome Project (formally finalized in 2001) brought about radical changes in technology (e.g. massively parallel DNA sequencing), most notably the ability to sequence entire genomes in a matter of days, not decades. In 2010, our group reported the complete genome, transcriptome, and methylome of a pair of discordant MS twins, representing the seventh and eighth whole human genomes ever sequenced, the first female, and naturally, the first MS genome.^17^ While thousands of differences were identified between the genomes (mostly in antibody and TCR genes) and an even larger number of differentially methylated (and therefore) expressed genes in helper T cells, the significance of those differences in a single pair was inconclusive. Yet, this effort (deposited in public databases) was a remarkable contribution to our collective understanding of MS genetics and paved the way for subsequent studies.

**Figure 1.**
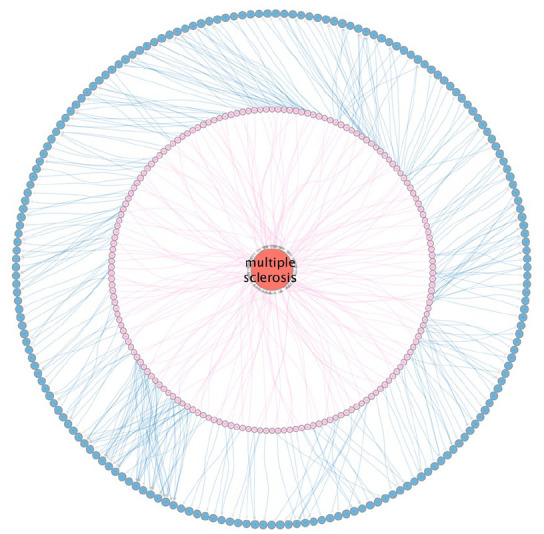
SPOKE representation of MS Genetics. The large red circle (node) in the middle represents MS. The inner circular arrangement of nodes connected by pink links (edges) represents the more than 200 genome-wide associated variants. The outer arrangement of blue nodes connected by blue edges represents the nearest or most likely affected genes to the associated variants.

The next milestone in MS genetics research was realized when samples from 9772 patients collected by 23 research groups working in 15 different countries were analyzed in a large GWAS, using a pool of 17,376 controls from the Wellcome Trust Case Control Consortium (WTCC). That study not only resulted in the identification of dozens of loci implicated in cytokine pathways, signal transduction, and molecules of immunological relevance but also those related to environmental risk factors such as vitamin D and the disease-modifying therapies such as natalizumab and daclizumab.^18^ As new technologies emerged, so did the opportunities to explore MS genetics in more profound ways. Using the ImmunoChip custom genotyping array, the IMSGC analyzed 14,498 individuals with MS and 24,091 healthy controls for 161,311 autosomal variants. This study identified 48 novel associations, taking the total to 110.^19^ With each study, an analysis of heritability was performed, and the results consistently indicated that a larger number of associations were still being missed due to their predicted modest effect sizes. To address this challenge, the IMSGC embarked on the ultimate effort to uncover all risk-associated variants in a new GWAS boasting 47,429 MS and 68,374 control subjects. This last effort resulted in the identification of 236 independent associations (36 of them within the HLA region), comprising the most complete genomic map of MS susceptibility to date (Figure 1).^20^

Understanding that, while additional associations may still exist, they would likely have very small effects (less than 5% increased risk per variant), the IMSGC turned to whether genetic variation influenced disease severity. Addressing this question required a different study design and a departure from the family-based and case–control studies of the past. To study severity, a cohort of 12,584 subjects was assembled for whom at least one measure of disease severity (determined by the Expanded Disability Scale Status, EDSS) was known. With the additional information on the time of diagnosis and the subject’s age, the age-related multiple sclerosis severity score (ARMSS) was computed and used as the primary study variable. This study identified the DYSF-ZNF638 locus as the first genome-wide significant association with MS severity.^21^ Remarkably, individuals with two copies of the associated allele had a shortening median time to requiring a walking aid of a median of 3.7 years and more cortical pathology in brain tissue.

Taken together, the association of 236 common variants with MS susceptibility and one variant with disease severity has paved the way to a complete understanding of MS pathogenesis and to facilitate the development of therapies that address specific dysregulated pathways. On the contrary, the partial heritability of the disease has also prompted efforts to understand the environmental component of MS.

## Environment

The fact that monozygotic twins are more concordant for MS than dizygotic twins supports one of the cornerstone hypotheses for a genetic contribution to MS risk.^22^ On the contrary, the fact that the concordance between monozygotic twins is less than 50% indicates a clear environmental contribution.^23,24^ Multiple environmental factors have been unequivocally linked to MS risk, including Epstein-Barr virus infection, smoking, and vitamin D deficiency.^25^ The advent of massively parallel DNA sequencing also spurred the new field of microbiome science, and with it, new associations with MS emerged.

The human gut contains trillions of microorganisms representing thousands of different species. Most of these are harmless (i.e. commensals) or even beneficial (fiber-degrading bacteria), while very few are capable of causing infectious diseases. However, the close proximity between microbes and immune cells in specialized regions of the gastrointestinal (GI) tract (e.g. Peyer patches, gut lamina propria) presents latent potential for immune system activation through various mechanisms.^26^

Some of the earliest work in this field was performed in mice and showed that the gut microbiota was necessary for experimental autoimmune encephalomyelitis (EAE) to develop, as antibiotic-treated animals were spared from it. Interestingly, GI monocolonization with a single bacterial species (segmented filamentous bacteria) was sufficient to restore EAE susceptibility, suggesting a close functional link between the gut and the immune system in this setting.

In 2016, the first human studies showing potential differences in the gut microbiome between individuals with and without MS were published.^27,28^ Shortly after, other human studies also identified differences between MS and controls, including pediatric cases.^29,30^

These pioneering studies showed that, like in other immune-related disorders, the gut microbiome should be considered part of the constellation of non-genetic factors influencing MS risk. However, unlike genetics, the interrogation of the gut microbiome is hampered by multiple external factors that could erode, confound, or, worse, invalidate study results. These factors (e.g. diet, geography, and use of disease-modifying therapy), in addition to small sample sizes, were responsible for the initial lack of consistency and replication among studies.

With initial support from the National MS Society and long-term philanthropic commitment, the international MS microbiome study (iMSMS) was established with the goal of providing the most comprehensive map of the MS microbiome and focused on minimizing those confounding factors.^31^ In order to achieve these goals, the iMSMS only recruits household MS control pairs, thus essentially controlling for diet, environment, and lifestyle. In its first effort, the iMSMS recruited and analyzed the gut microbiome of 128 pairs from 5 cities (San Francisco, New York, Boston, Edinburgh, and Buenos Aires). This study demonstrated that the largest confounder is household (followed by city) and confirmed that people who live in the same house eat a similar diet, reflected in a more similar microbiome than cross-house pairs.^32^ A follow-up study by the iMSMS included 576 pairs from 7 cities (adding Pittsburgh and San Sebastian to the previous list), which constitutes the most extensive microbiome study in MS to date.^33^ This study identified a significantly increased proportion of several microbes among individuals with MS, including *Akkermansia muciniphila, Ruthenibacterium lactatiformans, Hungatella hathewayi*, and *Eisenbergiella tayi*, and decreased *Faecalibacterium* *prausnitzii* and various *Blautia* species. Unlike previous reports, this iMSMS project used “shotgun” metagenomic sequencing, which, in addition to the presence of taxa, also allowed the identification of bacterial pathways (e.g. phytate degradation and pyruvate-producing) encoded in their genomes. Finally, the large number of participants also allowed for a sub-analysis to identify bacterial taxa associated with different treatment modalities.

Taken together, these results strongly support specific gut microbiome associations with MS risk, course and progression, and functional changes in response to treatment. These studies also highlight the complexity of MS pathogenesis, which, in addition to a genetic predisposition, also involves multiple environmental factors that could trigger or perpetuate the pathogenic process.

## The power of information

Correctly diagnosing, managing, and prognosticating MS require training, skill, and expertise. With more qualitative and quantitative tools available to the physician and researcher and a vast corpus of specialized information at their disposal, a new kind of aptitude will soon be necessary: multi-modal data integration. Even if genomic, proteomic, microbiome, and other sources of information are available, neurologists are still bound to only use imaging (magnetic resonance imaging (MRI)), clinical, and limited laboratory (e.g. oligoclonal bands) results to diagnose and manage the disease.

Under the new paradigm of multi-modal data integration, it will be possible to expand the characterization of any given patient using all their available information. However, this process will require a (likely computerized) system able to recall, link, and help interpret how those variables can be “assembled” into a unique model that best describes the patient in question. While current methods with the ability to integrate multiple variables and predict an outcome already exist (from statistical modeling to classical machine learning), those methods cannot explain how they arrive at their results (i.e. they are not explainable), representing potentially useful but “black box” solutions.

**Figure 2.**
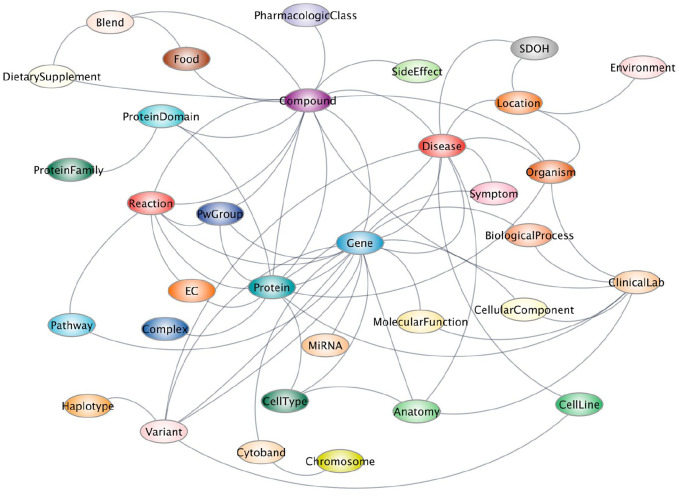
SPOKE metagraph. Each colored circle represents a type of node in SPOKE, and each gray line represents a type of relationship.

Knowledge graphs are vast networks of domain-specific, semantically connected concepts in a machine-readable environment. By downloading and connecting dozens of biomedical databases ranging from genetics to pharmacology to cell biology and more, our group created the scalable precision medicine open knowledge engine (SPOKE) graph.^34^ SPOKE contains tens of millions of concepts (from proteins, genes, and pathways to compounds, diseases, and symptoms) linked via more than a hundred million semantic relationships, thus constituting a machine-readable semi-complete representation of society’s collective biomedical knowledge (Figure 2). By capitalizing on advances in graph theory and artificial intelligence, SPOKE can be readily used to prioritize drugs for repurposing, identify novel drug targets, and interpret results from laboratory experiments, particularly those involving “omics” technologies.

Another area in which SPOKE has been used is integrating and interpreting information from a given patient. While not initially designed for research, access to electronic health records (EHRs) is becoming a powerful strategy to identify unexpected associations, mainly because of the sheer size of some of those data sets, which can reach tens of millions. In addition, research-oriented, population-based cohorts such as the UK Biobank^35^ and AllofUs^36^ provide deeply characterized data sets for computational scientists to develop and apply existing and novel algorithms to discover disease causes, predict diagnosis, response to exposures or treatments, etc. Several groups worldwide have taken advantage of these resources and reported discoveries ranging from intriguing to groundbreaking.^37^ Integrating patient clinical or research records with SPOKE via graph-traversing algorithms (some much like Google’s Page Rank) has the potential to increase performance and interpretability to the previously described approaches.^38^ This last point is not trivial, as any algorithm that classifies individuals into any category using health-related information is unlikely to be adopted by providers unless it can provide the biomedical basis for its recommendation.

In 2021, we reported that by implementing an embedding algorithm on the structured fields of 2.3 million EHR onto SPOKE and feeding the resulting features into a machine learning model, we were able to identify the ~5000 individuals with MS in that cohort. The SPOKE-based model could predict who would develop MS even using only the data from medical encounters in the primary care setting and years before the first formal MS diagnosis code was ever entered into the system.^39^ Such a model (which only included comorbidities, symptoms, labs, and medications) could be representative of an incipient trend in modern medicine, where more sophisticated approaches could consist of genomics, imaging, use of wearables, and more nuanced and valuable information buried in the notes. A key feature of these models is their explainability, as it is possible to identify through which nodes in the vast SPOKE semantic network information flows the most when analyzing individuals with MS and through which nodes information flows when analyzing individuals without the disease.

A similar approach has been applied to other chronic diseases, including Parkinson’s (PD)^40^ and Alzheimer’s (AD),^41^ with many others under investigation. While nodes such as depression, frequent use of antibiotics (likely related to urinary symptoms), fatigue, and specific immune genes and pathways were highly traversed by data from individuals about to develop MS, nodes representing tremor, constipation, and anosmia were highlighted in individuals before a PD diagnosis.

The vast amounts of information doctors are confronted with clearly exceed the human capacity to recall, relate, interpret, and react. While it is unlikely that computers and AI will replace humans in making patient-centered decisions, it is no secret that they are better suited to conducting systematic and consistent evaluations and considering multiple parallel possibilities that fit a given patient. The integration of these knowledge-based approaches with generative AI (like OpenAI’s Chat-GPT, Meta’s Llama, or Google’s Gemini) through methods like knowledge graph-based retrieval augmented generation (KG-RAG)^42^ will likely transform the doctor–patient relationship and the global healthcare system in the next few years.

## Conclusions

My 25-year-long career in MS research has been characterized by exploring new technologies, analysis methods, and overall collaborative work. My passion for untangling the complexities of MS encouraged me to explore other diseases and even other fields, such as physics, mathematics, and computer science, to learn and bring relevant aspects of those disciplines to my research. While these experiences nurtured me as a scientist, I genuinely believe that multi-disciplinary collaboration is the “secret sauce” of all great science. In particular, many disease-related aspects (like genetics, microbiome, etc.) are simply too great of a task for a single lab to tackle and will only be effectively solved through partnerships. Furthermore, as more and more information is generated for a given patient, its full integration into biologically aware models will enable the best possible care.

While there is still work to do, there has never been a time when more progress has been made than in the previous 25 years. It is my hope that through innovative research and collaborative work, the goal of stopping, restoring, and ending MS forever is now within reach.
